# Predicting collaborative practice between midwives and obstetricians: A regression analysis

**DOI:** 10.18332/ejm/192696

**Published:** 2024-09-24

**Authors:** Liesa Beier, Qendresa Thaqi, Ans Luyben, Nina Kimmich, Rahel Naef

**Affiliations:** 1 Department of Obstetrics, University Hospital Zurich, Zurich, Switzerland; 2 Department of Medical Sciences, Private University in the Principality of Liechtenstein, Triesen, Liechtenstein; 3 Institute for Implementation Science in Health Care, Faculty of Medicine, University of Zurich, Zurich, Switzerland; 4 Centre of Clinical Nursing Science, University Hospital Zurich, Zurich, Switzerland; 5 Department of Obstetrics, Lindenhofgruppe Bern, Bern, Switzerland; 6 Centre for Midwifery, Maternal and Perinatal Health, Bournemouth University, Bournemouth, United Kingdom; 7 University of Zurich, Zurich, Switzerland, Zurich, Switzerland

**Keywords:** physicians, hospital, obstetrics, midwifery, interprofessional collaboration, collaborative practice

## Abstract

**INTRODUCTION:**

Effective collaborative practice between midwives and obstetricians improves patient safety and obstetrical outcomes, but its implementation remains challenging. Therefore, its determinants need to be better understood. This study examined factors impacting collaborative practice (CP) between these professional groups.

**METHODS:**

This study was a cross-sectional survey that took place in Swiss hospital labor wards in 2021. Collaborative practice perceptions of 70 midwives (57.4% response rate) and 44 obstetricians (29.0% response rate) were assessed using the Interprofessional Collaboration Scale, with the score serving as the main outcome. A total of 13 individual, behavioral, and organizational predictors were analyzed by multiple linear regression.

**RESULTS:**

Participants rated collaborative practice with a median score of 3.1 (IQR: 2.8–3.4) out of a maximum score of 4.0. Results showed that five predictors significantly influenced collaborative practice: type of profession (β= -0.180; 95% CI: -0.296 – -0.040, p=0.011), trust/respect (β=0.343; 95% CI: 0.085–0.040, p=0.000), shared visions/goals (β=0.218; 95% CI: 0.030–0.204, p=0.009), workplace (β=0.253; 95% CI: 0.089–0.445, p=0.004) and shared power (β=0.163; 95% CI: 0.042–0.222, p=0.015). The model explained 66% of the variance (adjusted R2) in collaborative practice in labor wards.

**CONCLUSIONS:**

This study has identified key factors influencing CP in Swiss labor wards: workplace characteristics that require tailored CP models, and a power-sharing culture that fosters trust, respectful interactions and shared goals, requiring active exchange between midwives and obstetricians.

## INTRODUCTION

Effective collaborative practice (CP) between different professions is essential in maternity care to safeguard women’s and newborns’ well-being^1^. Collaborative practice is defined as ‘multiple health workers from different professional backgrounds working together with patients, families, caregivers, and communities to deliver the highest quality of care’^2^. Successful CP increases women’s safety and improves obstetrical outcomes due to less medical interventions and improved management of emergencies^1,3^.

Previous studies in the field of maternity care have highlighted the importance of CP. In the United States, The Joint Commission concluded in an evaluation of 47 adverse events (AEs) in maternity care, in which the problems in 72% of the cases were due to failure in teamwork and communication^1^. In 2022, the Final Ockenden Report published an investigation of 1592 AEs involving mothers and newborns in the United Kingdom. Cases from the year 1973 onward were reviewed by experts and by interviews with family and staff members. The report concluded that AEs could have been avoided in many cases and resulted from a lack of interprofessional communication and collaboration^3^.

Midwives and obstetricians are aware that the quality of CP is associated with AEs and directly impacts the care of women and newborns^4^. Both professions consider successful CP important and report that positive experiences in CP enhance their team spirit^4^. However, in a Dutch study, both professions scored low on mutual collaboration, with only 47% of the obstetricians and 44% of the hospital midwives being satisfied with CP within their team^5^. In Canada, 97% of the obstetricians and 100% of the midwives agreed that interprofessional relations could be improved.^6^

Previous studies have reported different care philosophies and ideologies among midwives and obstetricians. These were identified as fundamental contributing factors in CP^6-8^. A culture of ‘them and us’ was reported, limiting effective CP^3^. Further studies highlighted negative experiences concerning power imbalances, varying perceptions such as missing joint definitions of responsibilities, and hierarchical and competitive thinking^4,9^. Disrespectful behaviour^6,10^, a general lack of trust^11^, and a lack of conflict resolution strategies^9^ have also been found to hinder CP between midwives and obstetricians. On an organizational level, the absence of opportunities for exchange and discussion on a level playing field, as well as the general manner of communication, have been criticized on both sides^10,11^.

The current state of evidence clearly shows that CP is as important to midwives and obstetricians, but is often experienced in negative ways. The current implementation of CP in labor wards seems to be insufficient. Both professions express a need to increase CP in order to achieve positive birthing outcomes for women and their families^4,12^. Although the factors influencing CP have been previously investigated, their strength and magnitude are not well understood. Further, there is a general lack of research on CP, including both professional perspectives. In addition, it is strongly recommended that local, context-specific models for CP be developed not only for each country but also for every community and place of work, due to its multifactorial uniqueness^13^. Hence, research that builds on existing knowledge but in new cultural settings is needed. Even though CP has been investigated in several Western healthcare systems, to date, no studies have investigated the extent of CP between midwives and obstetricians in Switzerland. In Switzerland, over 96% of all births take place in hospitals. They are attended by midwives and obstetricians^14^, with obstetricians having a supervisory role over midwives^15^. Midwife-led births are rare.

Therefore, we conducted a cross-sectional survey study to assess Swiss midwives’ and obstetricians’ perceptions of current CP and examined the influence of individual, behavioral, and organizational predictors on the extent of CP.

## METHODS

### Study design and conceptual frame

A multi-center, cross-sectional survey investigated the CP between midwives and obstetricians. This study aimed to examine the impact of individual, behavioral, and organizational predictors on the CP between different professional groups in acute care labor wards.

**Figure 1:**
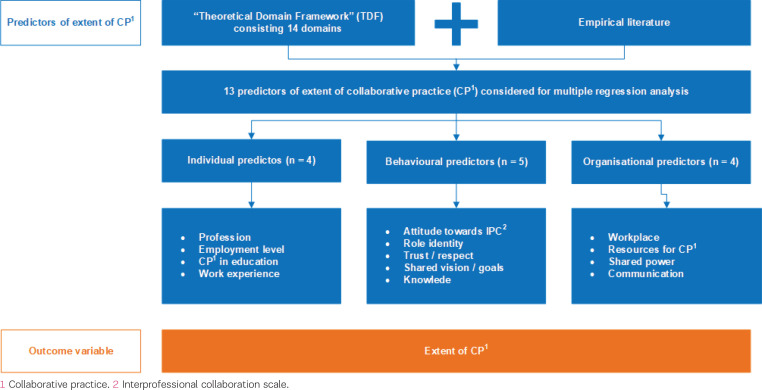
Conceptual study frame for regression analysis on collaborative practice

A combination of the Theoretical Domain Framework (TDF) and a review of empirical literature was the basis of the selection of factors investigated in this study and served as the conceptual frame (Figure 1). The TDF^16^ was chosen because it provides a theoretical basis for examining influences on individuals’ behavior, motivation, and skills in implementing certain evidence-based behaviors. It consists of 14 domains covering possible influencing factors on behavior. These factors were grouped on two levels: five behavioral predictors (attitudes, role identity, trust/respect, shared vision/goals, knowledge about the competencies of the other professional group) and four organizational predictors (place of work, resources for collaborative practice, shared power, communication structures). These predictors were expanded by seven individual predictors: age, gender, profession, employment level, work experience overall and on study labor wards, and whether collaborative practice has been part of their curriculum. It was hypothesized that individual, behavioral, and organizational factors influence CP to varying degrees. The STROBE guidelines for reporting cross-sectional studies were used^17^.

### Setting and participants

The study was conducted in labor wards of three acute care hospitals in the German-speaking part of Switzerland, which yearly serves between 1344 and 2582 women giving birth^18^. These hospitals were chosen because they treat a high proportion of high-risk women who need a closer CP between midwives and obstetricians.

Participating hospitals included a university-affiliated hospital with a full range of obstetrical care, including births from 23 gestational weeks and maternal and fetal (high) risk pregnancies (Workplace A), a large urban teaching hospital (Workplace B), and a regional hospital (Workplace C), which both take care of women from 32 gestational weeks onward^19^.

Participants were registered midwives and obstetricians working clinically on the participating labor wards for at least three months. Sufficient German language skills were required to complete the online survey. Student midwives and medical students were excluded from the study. A power calculation with G*Power showed that for a medium effect size (f^2^=0.15), with 13 predictors, a power of 0.8 with a significance level alpha <0.05, the minimum sample size for multiple regression was predicted to be 131 data sets^20^.

### Recruitment and data collection

Data collection occurred between August and November 2021 using REDCap^®^ (Vanderbilt University, Tennessee, USA), an electronic data capture system^21,22^. The survey consisted of four sections: 1) individual level (7 questions); 2) behavioral level (37 questions); 3) organizational level (8 questions); and 4) interprofessional collaboration (13 questions). The survey took 15–20 minutes to complete.

To recruit participants, the study was presented at each hospital by the first author. A local responsible person, such as a consultant midwife, senior physician, or clinical director, was identified to promote the study and encourage peers to participate. All 122 midwives and 152 obstetricians who met the inclusion criteria were invited to participate in the survey by email. Emails were sent out by the local person responsible for the study, and information about the study and a link to the online survey were included. Participants had a time window of 12 weeks to complete the survey. Three reminders were sent by email to all potential participants.

### Ethical considerations

The study was submitted to the responsible Ethics Committee of the Canton of Zurich, which waived the need for approval based on Swiss law, on 23 July 2021 (Req-2121-00827). Informed consent was obtained online by checking a box at the beginning of the survey.

### Measurement

Psychometrically validated instruments were used in the German language version and are presented below^23-25^. Written permission was obtained from the authors of the German versions before the study. For each predictor for which we could not identify a suitable, valid instrument, items were formulated by the first author and then discussed with the research team. Questions were pretested with three midwives. All testers rated the questions as understandable and clear.

### Outcome variable

The 13-item German version of the Interprofessional Collaboration Scale (IPC) was used to assess the current perception of CP between the professions^23^. The IPC was developed to analyze CP’s different professional group-related perspectives. It consists of three subscales, which were identified as key factors in the validation process of the original scale: ‘Communication’, ‘Accommodation’, and ‘Isolation’. The two subscales, ‘Communication’ and ‘Accommodation’, address important aspects of the search for understanding and contain five items each. The subscale ‘Isolation’ examines possible strained relations between the professional groups with three items^26^. Item responses are rated on a 4-point Likert scale (score range: 1= ‘strongly disagree’, to 4= ‘strongly agree’). High median scores on the overall scale reflect a positive perception of CP. Cronbach’s a in the current sample was 0.89 for the total scale and between 0.62 and 0.80 for its subscales (communication a=0.89, accommodation a=0.80, and isolation a=0.62), showing high internal consistency except for the isolation subscale^27^.

### Predictive variables

*Individual level*

Individual information was collected using seven closed questions, including age (years), gender (male/female), profession (midwife/obstetrician), employment level (percent), work experience overall and in the study labor wards (years and months), and whether CP has been part of their curriculum (yes/no).

*Behavioral level*

Five predictors were assessed pertaining to obstetrician and midwife behavior. First, attitudes toward collaborative practice were assessed using the German version of the Jefferson Scale of Attitudes towards Interprofessional Collaboration (JeffSATIC)^24^. High scores imply an open attitude towards interprofessional collaboration (score range: 20–140)^28^. In the current sample, Cronbach’s a of the JeffSATIC was 0.69^27^.

The German version of the Extended Professional Identity Scale (EPIS) was used to measure interprofessional role identity^25^. The EPIS has three subscales covering interprofessional belonging, interprofessional commitment, and interprofessional beliefs. High scores indicate a high interprofessional role identity (range: 12–60). The German version of the EPIS scale is currently validated. Cronbach’s α in the current sample was 0.88^27^.

Trust and respect, shared vision and goals, and knowledge were assessed with self-developed questions (Table 1).

**Table 1:** Overview measures of behavioral and organizational independent variables for regression analysis on collaborative practice among registered midwives and obstetricians working clinically in labor wards of three acute care hospitals, August–November 2021, Switzerland (N=114)

*Study endpoints*	*Measures*	*Items*	*Score range^a^*	*Score type*	*Cronbach’s alpha^b^*
**Independent variables on behavioral level**					
**Attitudes towards IPC**	Jefferson Scale of Attitudes towards Interprofessional Collaboration (JeffSATIC)	20	1–7	score	0.69
**Role identity**	Extended Professional Identity Scale (EPIS)	12	1–5	mean	0.88
Trust/respect*	‘We treat each other with respect in interprofessional collaboration.’ ‘We show trust in each other in interprofessional collaboration.’	2	1–5	mean	0.89
Shared vision/goals*	‘We share similar goals in obstetric care.’ ‘We share similar visions of the implementation of these goals in obstetric care.’	2	1–5	mean	0.87
Knowledge*	‘I know the competency and responsibility profile of the other professional group.’	1	1–5	mean	NA
**Independent variables on organizational level**					
**Workplace^§^**	A, B, C	1	NA	NA	NA
Resources for collaborative practice*	‘In our daily practice, we have sufficient resources to provide patient care as an interprofessional team.’ ‘In our daily practice, we have sufficient resources to develop a culture of interprofessional collaboration.’	2	1–5	mean	0.80
Shared power*^c^	‘I ask the other professional group about their expectations regarding the extent of my involvement in the decision-making process of obstetric care.’ ‘I discuss with the other professional group the extent to which I would like to be involved in the planning and implementation of the obstetric procedure.’ ‘I suggest to the other professional group approaches to care which I consider useful.’ ‘I discuss areas of agreement and disagreement with the other professional group in order to develop mutually agreeable goals of obstetric care.’	4	1–5	mean	0.58
Communication*	‘In our organization, we have sufficient forums that enable interprofessional communication.’	1	1–5	mean	NA

a 1=strongly disagree to 5 or 7=strongly agree.

b Related to the current sample.

c Adapted from the German version of the ‘Collaborative Practice Scale’ (collaborative practice).

* Self-developed questions. NA: not applicable.

§ Workplace A: university-affiliated hospital with a full range of obstetrical care. Workplace B: a large urban teaching hospital. Workplace C: a regional hospital.

*Organizational level*

At the organizational level, the workplace (A, B, C), resources for CP, shared power, and communication were assessed. Four questions were adapted from the German Collaborative Practice Scale version to measure a sense of shared power^29,30^. Table 1 provides details on all self-developed variables.

### Data analysis

Survey data were exported from REDCap^®^^21,22^ into SPSS version 26 (IBM Corp., Armonk, New York, USA)^31^, which was used for data analysis. Data were checked for plausibility and completeness using duplicates, outliers, or response trends; 27 (19.15%) incomplete responses were removed. Due to inaccurate information on age and professional experience, the answers of one participant were excluded from the analysis.

Data distribution was tested using skewness, kurtosis, and Shapiro-Wilk. Data for the outcome variable were normally distributed, but not for the independent variables, as assessed by the Shapiro-Wilk-test, p<0.05. Variance homogeneity in profession and workplace was assessed using Levene’s test, which showed that equal variances could be assumed, p>0.05.

Descriptive statistics were used to present participant characteristics and study endpoints according to professional groups. Due to non-normal data distribution, results are presented by median and interquartile range.

The influence of individual, behavioral, and organizational factors (independent variables) on the CP (outcome variable) was analyzed using multiple linear regression analysis. All 13 independent variables were included simultaneously (ENTER method) in the model. To test the influence of the place of work, the variable ‘workplace’ was dummy-coded and calculated twice using different reference categories. Assumptions for multiple linear regression analysis were tested and met. There was linearity, as assessed by scatter plots. Analysis of collinearity statistics showed VIF scores were well below 10 and tolerance scores above 0.2. Residuals were independent, as assessed by a Durbin-Watson statistic of 2.082. The plot of standardized residuals versus standardized predicted values showed no obvious signs of funneling, suggesting the assumption of homoscedasticity has been met. The P-P plot for the model suggested the values of the residuals are normally distributed. Cook’s distance values were <1, suggesting individual cases did not influence the model. Effect sizes for multiple linear regression analyses were calculated according to Cohen (f^2^=0.02 small effect size, f^2^=0.15 medium effect size, and f^2^=0.35 large effect size)^32^. For all statistical analyses, a p<0.05 was considered the criterion for significance.

## RESULTS

### Participants’ characteristics and perceptions of collaborative practice

A total of 114 (41.6%) of 274 invited professionals completed the survey; 44 were returned by obstetricians (response rate 29.0%) and 70 by midwives (response rate 57.4%). The largest number of participants took part in the survey from Workplace A (n=59; 51.8%), followed by Workplace B (n=28; 24.6%) and Workplace C (n=27; 23.7%). The mean age of participants was 36 years (IQR: 29–50), and the gender breakdown was 107 females (93.9%) and 7 (6.1%) males. The mean work experience in the profession of all participants was 9 years (IQR: 3.0–20.0), 9.25 years for midwives (IQR: 3.0–23.5), and 7.5 years for obstetricians (IQR: 3.8–15.0).

Across all participants, CP was rated with a median score of 3.1 (IQR: 2.8–3.4) out of a possible score of 4. Professions differed in their perception of CP, with obstetricians appraising CP with midwives at 3.4, whereas midwives perceived CP with obstetricians as 2.9. The communication subscale was rated highest with a median score of 3.2 (IQR: 2.8–3.6), followed by the accommodation with 3.0 (IQR: 2.8–3.4), and isolation subscales with 3.0 (IQR: 2.7–3.3). Table 2 details the characteristics of the study cohort and study endpoints.

**Table 2:** Characteristics of the registered midwives and obstetricians working clinically in labor wards of three acute care hospitals, August–November 2021, Switzerland (N=114)

*Characteristics*	*All (N=114) Median (IQR)*	*Obstetricians (N=44) Median (IQR)*	*Midwives (N=70) Median (IQR)*
**Individual level**			
**Age** (years)	36.0 (29.0–50.0)	34.5 (31.0–44.8)	36.5 (28.0–53.0)
**Female**, n (%)	107 (93.9)	37 (84.1)	70 (100)
**Percent employment level**	80.0 (70.0–100)	100 (80.0–100)	80.0 (60.0–90.0)
**Work experience** (years)			
In profession	9.0 (3.0–20.0)	7.5 (3.8–15)	9.3 (3–23.5)
At workplace	5.0 (2.0–13.0)	3.0 (1.5–8.0)	5.6 (2.5–14.0)
**CP in education** (yes), n (%)	54 (47.4)	15 (34.1)	39 (55.7)
**Behavioral level**			
Attitudes towards IPC	123.0 (115.8–128.0)	121.0 (110.3–129)	123.0 (117.8–128.0)
Role identity	4.5 (4.0–4.8)	4.5 (4.0–4.8)	4.5 (4.1–4.7)
Trust/respect	4 .0 (4.0–4.8)	5.0 (4.0–5.0)	4.0 (3.0–4.0)
Shared vision/goals	4.0 (3.0–4.0)	4.0 (4.0–4.5)	3.5 (3.0–4.0)
Knowledge	4.0 (4.0–5.0)	4.0 (4.0–5.0)	4.0 (4.0–5.0)
**Organizational level**			
**Workplace^§^**, n (%)			
A	59 (51.8)	28 (47.5)	31 (52.5)
B	28 (24.6)	9 (32.1)	19 (67.9)
C	27 (23.7)	7 (25.9)	20 (74.1)
**Resources for CP**	3.5 (2.5–4.0)	4.0 (3.0–4.4)	3.0 (2.0–4.0)
**Shared power**	3.8 (3.4–4.0)	4.0 (3.5–4.3)	3.8 (3.25–4.0)
**Communication**	4.0 (2.0–5.0)	4.0 (3.0–5.0)	3.0 (2.0–4.0)
**Extent of CP**			
CP	3.1 (2.8–3.4)	3.4 (3.1–3.7)	2.9 (2.7–3.2)
Communication	3.2 (2.8–3.5)	3.4 (3.2–3.8)	3.0 (2.8–3.2)
Accommodation	3.0 (2.8–3.4)	3.2 (3–3.8)	3.0 (2.6–3.2)
Isolation	3.0 (2.7–3.3)	3.3 (3–3.7)	2.7 (2.3–3.0)

IPC: Interprofessional Collaboration Scale. IPC mean score ranging from 1= ‘strongly disagree’ to 4= ‘strongly agree’. CP: collaborative practice. IQR: interquartile range.

§ Workplace A: university-affiliated hospital with a full range of obstetrical care. Workplace B: a large urban teaching hospital. Workplace C: a regional hospital.

### Influence of individual, behavioral, and organizational predictors on the collaborative practice

The multiple linear regression showed that perceptions of CP are significantly influenced by individual, behavioral, and organizational predictors (B=1.187; p<0.001) (Table 3). According to Cohen, the model explained 66% of the variance (adjusted R^2^) in professionals` perceptions of CP, with a very strong effect (f^2^=1.94)^32^. Of the 13 analyzed predictors, five were found to have a statistically significant influence on the CP (Table 3).

**Table 3:** Regression model on collaborative practice among registered midwives and obstetricians working clinically on labor wards of three acute care hospitals, August–November 2021, Switzerland (N=114)

*Independent variables*	*B*	*β*	*t*	*p*	*95% CI*	
					*Lower*	*Upper*
**Constant**	1.19		2.22			
**Individual level**						
Profession^a^	-0.17	-0.18	-2.6	0.011**	-0.3	-0.04
Employment level	0.00	0.02	0.23	0.821	-0.003	0.004
CP in education	-0.08	-0.09	-1.33	0.188	-0.21	0.04
Work experience in profession	-0.01	-0.13	-1.94	0.056	-0.01	0.00
**Behavioral level**						
Attitude towards IPC^b^	0.01	0.11	1.68	0.096	-0.001	0.01
Role identity	-0.04	-0.03	-0.5	0.620	-0.001	0.01
Trust/respect	0.17	0.34	3.99	0.000***	0.09	0.26
Shared vision/goals	0.12	0.22	2.68	0.009***	0.03	0.20
Knowledge	-0.003	-0.01	-0.09	0.927	-0.08	0.07
**Organizational level**						
Resources for CP	0.04	0.09	1.17	0.243	-0.003	0.11
Shared power	0.12	0.16	2.47	0.015*	0.02	0.22
Communication	0.03	0.08	0.97	0.335	-0.03	0.08
**Dummy coded variables^§^**						
Workplace A vs Workplace C	0.02	0.19	0.20	0.841	-0.16	0.19
Workplace B vs Workplace C	0.27	0.25	2.98	0.004***	0.09	0.45
Workplace B vs Workplace A	0.25	0.24	3.38	0.001***	0.10	0.4

F (14, 98)=16.441, p<0.001, n=112, R^2^=0.70, adjusted R^2^=0.66, f^2^=1.94. Independent variable: extent of collaborative practice (collaborative practice mean score). B: unstandardized coefficient. β: standardized coefficient.

a 0 = obstetrician, 1 = midwife. CP: collaborative practice.

b The Jefferson Scale of Attitudes towards Interprofessional Collaboration.

§ Workplace A: university-affiliated hospital with a full range of obstetrical care. Workplace B: a large urban teaching hospital. Workplace C: a regional hospital.

* p<0.05,

** p<0.01,

*** p<0.001.

Among the five individual predictors, a significant influence was found for ‘type of profession’ (β= -0.18; 95% CI: -0.3 – -0.04, p=0.011) and ‘place of work’ (β=0.25; 95% CI: 0.09–0.45, p=0.004). Regarding the ‘type of profession’, CP decreased by -0.17 points when changing from obstetrician to midwife type of profession (β= -0.18; 95% CI: -0.3 – -0.04, p=0.011). Midwives rated the CP with obstetricians lower than obstetricians rated the CP with midwives. Compared to Workplace C, professionals from Workplace A and Workplace B perceived a more positive CP by 0.02 points (β=0.19; 95% CI: -0.16–0.19, p=0.841) and 0.27 points (β=0.25; 95% CI: 0.09–0.45, p=0.004), respectively. No statistically significant influence was found for the remaining individual predictors (employment level, having had CP in education, work experience in the profession) (Table 3).

On the behavioral level, two of the five predictors were statistically significant: ‘trust/respect’ and ‘shared vision/goals’. CP increased by 0.17 points (β=0.34; 95% CI: 0.09 –0.26, p=0.001) with higher perceptions of trust/respect, and by 0.12 points with more positive perceptions of shared visions/goals (β=0.22; 95% CI: 0.03–0.20, p=0.009). The other organizational predictors were not statistically significant (attitudes towards IPC, role identity, knowledge) (Table 3).

The results at the organizational level showed that one of the three predictors had a statistically significant influence on CP. A sense of ‘shared power’ significantly increased CP by 0.123 points (β=0.163; 95% CI: 0.024–0.222, p=0.015). For ‘resources for CP’ and ‘communication’ no statistically significant influence on CP was found (Table 3).

## DISCUSSION

This study investigated CP between midwives and obstetricians in acute care labor wards in the Swiss health system’s context, using data from both professional groups. We found that CP is best predicted by combining objective aspects, such as ‘profession’ and ‘place of work’, and self-perceived aspects of CP, such as ‘trust/respect’, ‘shared power’, or ‘shared vision/goals’. These significant predictors were able to explain two-thirds of the adjusted variance in CP.

This study makes three notable contributions to the existing knowledge of CP in labor wards. First, it contributes knowledge from a new cultural healthcare context, confirming previous research findings and showing that CP needs to be improved at various levels in Switzerland. Second, it involves both professional groups involved in CP in maternity care, allowing a more comprehensive understanding of collaborative maternity care and showing that midwives need more support than obstetricians in developing CP. Third, it is the first study to examine CP using regression analysis, thereby extending our understanding its multifaceted determinants, suggesting that different strategies at several levels are needed to promote interprofessional maternity care. Our findings add to previous research from other countries and healthcare settings, which identified similar CP determinants. However, in contrast to other studies, other influencing factors, such as ‘communication’, ‘resources for CP’, ‘knowledge’, and ‘CP in education’, were not significant predictors in our study.

In our sample, current CP was generally rated to be effective by both midwives and obstetricians. However, obstetricians rated CP with midwives higher than midwives rated CP with obstetricians. This is in line with a previous study from the Netherlands^5^, which also showed lower ratings in midwives compared to physicians. Qualitative studies have reported that midwives perceived a basic willingness of obstetricians to collaborate^4,7^, while obstetricians perceived midwives as reluctant to collaborate and felt viewed as ‘the enemy’^12^. Although obstetricians wished to increase CP, midwives reported being less confident in the future, enhanced CP^5^. Our findings, together with others, suggest that midwives perceive CP with obstetricians as less effective than vice versa. Based on our study, we are unable to identify explanations for this difference between professional groups. Previous studies have shown that professional groups’ definitions and understanding of successful CP vary^5,7^. A need to put together the viewpoints of the professions to create a general consensus among professional groups on the meaning of CP has been proposed.^7^ Our results suggest that in the Swiss healthcare context, it is vital for midwives and obstetricians to develop a shared understanding of effective CP and clarify mutual expectations for success.

The workplace was found to be another significant predictor of CP. This finding is consistent with previous studies, in which the development of adapted, specific models of CP is recommended due to the uniqueness of each place of work^13^. In our study, it was possible that the level of care explains the difference, as the hospitals differed in the kind of care they provided regarding the weeks of gestation and the accompanying maternal and fetal risk factors and diseases. A previous study found that the level of CP depends on the underlying model of care^33^. Qualitative research indicated that the institutional culture should emphasize effective teamwork to strengthen CP in the different models of care^33^. Therefore, we recommend that clinical leaders in individual institutions actively promote exchange between professional groups to define and shape CP within their hospital.

Characteristics of the workplace culture, such as self-perceived aspects of ‘trust/respect’, ‘shared power’, and ‘shared vision/goals’, significantly predicted perceptions of CP. Several international studies have highlighted trust and respect among midwives and obstetricians as factors significantly enhancing CP^11^. In contrast, disrespectful behavior and a general lack of trust have hindered CP between professional groups^6,10^ as both reported a general lack of trust in each other^6,11^. While midwives perceived a lack of trust in their skills^6^, obstetricians expressed frustration about midwives who did not trust that they had the best intentions for women giving birth^11^. Furthermore, both sides perceived the other professional group as disrespectful^6,9,10^. In the United Kingdom, 75% of junior doctors reported experiences of disrespectful, unprofessional behavior from midwives towards them^10^. In contrast, another study described midwives’ experiences of disrespect from obstetricians, especially towards newly qualified midwives^9^. Authors of several studies have shown that both professional groups long for more mutual trust and respect^5,6,11,33^. In our study, both professional groups viewed trust and respect as essential factors for successful CP. Institutions should, therefore, aim to improve interprofessional relationships based on mutual trust in skills, intentions, and respect in interactions.

We also identified a statistically significant influence of ‘shared power’ on CP. Both professional groups have experienced negative experiences concerning hierarchies, hierarchical thinking, and power imbalances^4^. An Australian study found that negative interactions occurred when a hierarchical model of medical dominance existed^4^. In our setting, this result might be explained by a similar prevailing hierarchical structure, as obstetricians are defined to be ultimately responsible for decision-making during birth^15^. In Norway, midwives working in a hospital scored significantly lower on the factor ‘autonomous professional role’ than midwives not working in a hospital^34^. A Belgian study also reported greater professional autonomy among independent midwives than hospital midwives^35^. Midwives further described the experience of authoritative behavior from obstetricians toward them and think that obstetricians see themselves as ranked above them^6^. Obstetricians expressed a sense of responsibility for their position in the formal hierarchy and described the problems of working as junior doctors with senior midwives^8^. Furthermore, responsibilities and competencies were often reported to be unclear or insufficiently defined^6,7,11^. Such lack of clarity, combined with negative experiences, leads to divergent needs for midwifery autonomy and defensive territorial attitudes between professional groups rather than working together to improve CP^11,12^. While midwives desired autonomy, some studies reported strategies for working independently without obstetrician intervention^11^. In contrast, obstetricians felt excluded and sought ways to improve CP and engage with the ‘midwifery world’ using various strategies^12^. Our results underline that clarity of roles and shared decisionmaking are necessary key factors in sharing power and thus enhancing CP.

We further found that ‘shared vision/goals’ significantly predicted CP. Differing philosophies of a ‘good birth experience’ between midwives and obstetricians limit effective collaboration, with both professional groups expressing a desire for better collaboration and integrated models of care^3^. In a survey study, significantly more obstetricians stated that they considered childbirth to be a dangerous process, possibly needing interventions, while midwives wanted to do everything possible to protect the physiological process of birth^6,12^. Having different views on a situation was cited by Wahlberg et al.^8^ as causing dysfunctional CP. Therefore, shared visions and role clarity have been proposed as essential principals in CP^36^. Round tables have been suggested for shared decision-making to achieve a common birthing vision^4^. We have identified that shared vision and goals in CP are important in Switzerland. We, therefore, recommend building a caring culture of shared power and shared vision/goals. This could be achieved through a clear division of roles, joint training, and further education. Joint training of midwifery and medical students could provide a shared vision before the respective philosophies become embedded, promoting effective CP.

All other individual, behavioral, and organizational influences were not statistically significant in our sample. Regarding the EPIS and JeffSATIC scales^24,25,28^, both instruments showed significant and non-significant results for group differences in studies investigating attitudes toward interprofessional collaboration and interprofessional role identity^24,25^. To our knowledge, the instruments have not been used for midwives and obstetricians, which limits the comparability of the results. The remaining non-significant factors, such as ‘communication’, ‘resources for CP’, ‘knowledge’, and ‘CP in education’ have been investigated in previous studies, mostly using qualitative methods, which have shown that these factors are important for both professional groups^7,9,11,33^. Compared to previous studies, we included multiple factors influencing CP and used multiple validated and non-validated instruments in our study. The combination of multiple predictors and instruments may have meant that factors identified as influential in the qualitative studies were not significant in our regression.

More research is needed to evaluate perceptions of collaborative practice among midwives and obstetricians in various Swiss maternity settings and other health systems and to understand the workplace specificities. Future studies will be necessary to investigate the strategies needed to improve and foster a culture of CP. In addition, an in-depth exploration of obstetricians’ experiences and perspectives is needed, as these are underrepresented in the research literature.

### Limitations

The current study has several limitations. First, a convenience sampling method and the different levels of care provided by the participating institutions might have affected the external study validity and limited the results’ generalization. Second, a selection bias might have affected this survey. Midwives and obstetricians with strong opinions on CP, favorable or unfavorable, could have had a pronounced interest in responding to the survey, which might have skewed the results. Third, we did not reach the pre-calculated sample size, missing 17 participants. Therefore, non-significant predictors in the multiple regression analysis may be a consequence of the missing statistical power. Nonetheless, we were able to identify significant predictors of CP. Fourth, internal validity could be negatively affected by applying several self-developed, non-validated items despite the fact that the self-developed questions showed good internal consistency in our sample. Moreover, whenever possible, validated and psychometrically sound instruments were used^23-25^.

## CONCLUSIONS

This study examined collaborative practice (CP) between midwives and obstetricians in Swiss acute labor wards. It identified several key factors influencing CP. First, the specific characteristics of the workplace significantly impact CP, making it essential to tailor CP models to the unique environment and culture of each maternity care setting. Second, the culture of shared power between professional groups promotes CP by fostering trust, respectful interactions, and shared goals, and active exchange between midwives and obstetricians is required to build mutual trust and respect. Third, midwives need targeted support and resources to enhance their CP skills and fulfill their desire for effective CP.

## Data Availability

The data supporting this research are available from the authors on reasonable request.
